# Monkeypox Virus Occurrence in Wastewater Environment and Its Correlation with Incidence Cases of Mpox: A Systematic Review and Meta-Analytic Study

**DOI:** 10.3390/v17030308

**Published:** 2025-02-24

**Authors:** Cornelius A. Omatola, Ropo E. Ogunsakin, Ademola O. Olaniran, Sheena Kumari

**Affiliations:** 1 Institute for Water and Wastewater Technology, Durban University of Technology, P.O. Box 1334, Durban 4000, South Africa; sheenak1@dut.ac.za; 2 Department of Microbiology, Kogi State University, Anyigba 272102, Nigeria; 3 School of Health Systems and Public Health, Faculty of Health Sciences, University of Pretoria, Pretoria 0084, South Africa; oreropo@gmail.com; 4 Discipline of Microbiology, School of Life Sciences, College of Agriculture, Engineering and Science, Westville Campus, University of KwaZulu-Natal, Private Bag X54001, Durban 4000, South Africa

**Keywords:** mpox virus, mpox, incidence cases, wastewater surveillance

## Abstract

The COVID-19 pandemic has increased the interest in the use of wastewater-based surveillance (WBS) strategy for infectious disease monitoring, especially when clinical cases are underreported. The excretion of monkey virus (MPXV) in the feces of both symptomatic and preclinical individuals has further driven the interest in WBS applicability to MPXV monitoring in wastewater to support its mitigation efforts. We performed a systematic review with meta-analysis, using six databases to assess MPXV detection in wastewater. We performed a random-effects model meta-analysis to calculate the pooled prevalence at a 95% confidence interval (95% CI). Also, we carried out a subgroup analysis according to the country regions and a sensitivity analysis excluding studies classified as having a high risk of bias. The overall MPXV positivity rate in wastewater was estimated at 22% (95% CI: 14−30%; *I*^2^ = 94.8%), with more detection rate in North America (26%, 95% CI: 8–43%) compared to Europe and Asia (22%, 95% CI: 12–31%). The MPXV detection rate was significantly higher in 2022 studies (22%, 95% CI: 13–31%) compared to 2023 (19%, 95% CI: 14–25%). The real-time PCR platform significantly detected more MPXV (24%, 95% CI: 14–34%) than the digital droplet PCR-based studies (17%, 95% CI: 4–31%), which was used less frequently. Viral concentration with centrifugation procedure indicated higher detection rates (21%, 95% CI: 10–33%) than other known sample concentration protocols. Generally, MPXV detection rates in wastewater samples strongly correlate with incidence cases of mpox (range of R = 0.78–0.94; *p* < 0.05). Findings from this study suggest that WBS of MPXV could be employed as an epidemiological early warning tool for disease monitoring and mpox outbreak prediction similar to the clinical case-based surveillance strategies.

## 1. Introduction

Monkeypox virus (MPXV) is an enveloped, double-stranded DNA virus in the genus *Orthopoxvirus* and family *Poxviridae.* Two distinct genetic clades of MPXV, namely, clade I (previously known as Congo Basin clade) and clade II (previously referred to as the West African clade) have been characterized. Clade I and clade II have been further typed as subclades, Ia and Ib and subclades, IIa and IIb, respectively [1]. MPXV causes mpox (previously called monkeypox) and since May 2022, the dynamics of the worldwide spread of the disease has increased, especially in previously unrecognized countries [2]. Since the first recognition of human mpox cases in 1970, the natural host reservoir has remained unclear, although numerous mammalian species including humans are susceptible [3]. Over the past five decades, the Central and West Africa region remain the regions with a significant burden of human mpox cases [4]. On 14 August 2024, the rising trend in ongoing global mpox outbreaks, especially in affected African countries, prompted the declaration of mpox by the World Health Organization (WHO) as a disease of Public Health Emergency of International Concern (PHEIC) [5]. The upsurge in morbidity and deaths, especially among young children, has been attributed to the stoppage of the smallpox vaccine and high rates of malnutrition in regions with significant disease burdens [6,7].

The current state of mpox is worrisome as a result of the change in the transmission dynamics, a high death rate, difficulty in accessing medical countermeasures, and the economic, social, and health impacts of the disease [5]. The changing epidemiology and the enormous threat posed to economic security and health by the mpox outbreak highlight the urgent need for close monitoring of the disease in both endemic and non-endemic areas. However, inadequate biosafety laboratory equipment in low resource areas, poor awareness of clinician and training levels, and the potential for subclinical conditions may limit clinical testing [8]. Over many years, wastewater-based surveillance (WBS) strategy has been used to track pathogens transmitted via water or fecal–oral route, providing a sensitive and reliable epidemiological monitoring indicator about pathogens circulating in a community. Unlike clinical testing, WBS offers a holistic, real-time, and low-cost disease monitoring approach, particularly for diseases that are not easy to monitor through a case-based surveillance strategy [9].

Infectious agents shed in urine, feces, or through respiratory secretions and sloughing of skin usually find their way into the wastewater treatment systems. Thus, raw wastewater acts as a composite biological sample and its nature has rapidly promoted the establishment of WBS as a public health tool to monitor trends in infectious diseases such as polio and COVID-19 in the community [9]. The excretion of MPXV in the feces of infected animals [10] and in feces, semen, saliva, urine, and skin lesions from infected humans [11] have been documented, supporting the potential benefit of WBS as a virus tracking tool for MPXV in wastewater environments. Notably, the approach could potentially capture both asymptomatic and/or subclinical cases and generate early warning signals of imminent mpox outbreaks in a community with inadequate access to healthcare [12]. In an outbreak investigation of mpox among animal models, MPXV DNA was detected in feces and urine of asymptomatic and symptomatic wild chimpanzees. In the former, the detected viral DNA in feces spanned a period of 12 days before the first symptoms visibly appear in the body [10]. In a study conducted among men in a Belgian sexual health clinic, three asymptomatic mpox cases were identified and results of clinical examination 21–37 days post MPXV-DNA detection indicated that they were free of clinical signs throughout [13]. Findings from these studies suggest that certain mpox cases may be undiagnosed and that clinical case testing and quarantining of symptomatic individuals may not be sufficient to contain the outbreak.

To our knowledge, there are currently no meta-analytic studies exploring the global epidemiology of MPXV in wastewater environments and its correlation with mpox cases. During the COVID-19 pandemic, genomic concentrations of SARS-CoV-2 in wastewater were strongly correlated with clinical case incidence [9,14], increasing the confidence in WBS as a monitoring tool that could guide public health response to disease outbreaks. Nevertheless, there needs to be more comprehensive information about the occurrence of MPXV in wastewater and the number of MPXV-infected persons during the same period. Thus, we reviewed several articles published globally between 2022 and 2024 and summarized the surveillance data from wastewater-based studies on MPXV and the corresponding mpox cases to estimate the overall prevalence for MPXV across different regions, draw a possible correlation between rates of detection in wastewater and number of individuals infected with the virus, and to further shed light on important data gaps that could inform future epidemiological research.

## 2. Methods

### 2.1. Study Design and Search Strategy

This systematic review was conducted according to the Preferred Reporting Items for Systematic Reviews and Meta-Analyses (PRISMA) guidelines. On 8 October 2024, six databases (PubMed, Google Scholar, Scopus, Science Direct, Wiley Online Library, and Web of Science) were systematically searched for peer-reviewed articles from inception to November 2024. The keywords string pattern used in the database search were “Monkeypox virus” OR “mpox virus” AND “wastewater” OR “sewage”, “Mpox virus” AND “wastewater” AND “mpox”, “environmental surveillance” AND “Mpox virus” AND “mpox”. In addition, hand searching and screening for relevant publications was carried out on all the references in the identified articles.

### 2.2. Inclusion and Exclusion of Studies

We included articles that provided outcome indicators regarding MPXV detection in wastewater, such as MPXV detection rate, genomic DNA concentration, and mpox cases during the surveillance period. To be included, studies must have employed real-time quantitative polymerase chain reaction (qPCR) alone or in combination with digital droplet polymerase chain reaction (ddPCR) for MPXV detection and quantification in the wastewater samples. Included articles were reported in English only and the sampling campaign must have taken place continuously for ≥3 months. In cases where studies were duplicated, only the articles with comprehensive details were included. Further, where the same study resulted in multiple publications, we only retrieved data from the available article that is most recent. We excluded articles in which the type of wastewater was not raw wastewater. Also, publications with no accessible MPXV-positive data and less than a 3-month monitoring period were excluded.

### 2.3. Study Selection

After completing the search strategy, the first author collected all the search results into a single Endnote library, which was used to remove all duplicates among the various databases considered in the search strategy. Two independent reviewers (C.A.O. and R.E.O.) screened the articles’ titles and abstracts for eligibility. Sequel to the elimination of irrelevant literature, the full texts of potentially eligible articles were further screened by two reviewers (C.A.O. and R.E.O.) and differences were resolved based on consensus between the investigators (C.A.O. and R.E.O.). Finally, all included studies were grouped and prepared for data extraction.

### 2.4. Data Extraction

The two investigators (C.A.O. and R.E.O.) who assessed the full texts extracted the data from all eligible articles into a working Microsoft Office Excel database. Information retrieved from the studies included details of the author, country where study was conducted, region (based on the United Nations classification or demarcation), sampling period and strategy, total number of samples collected, method of viral recovery or concentration, PCR method employed, recovery efficiency, cycle threshold (CT) values, primary outcome indicators (MPXV DNA concentration range, MPXV detection rate), and other indicators (number of total mpox cases during sampling period), where available.

### 2.5. Risk of Bias Assessment

The methodological qualities of each included study were evaluated by two independent reviewers (C.A.O. and R.E.O.) using a modified 11-item checklist developed by the Agency for Healthcare Research and Quality (https://effectivehealthcare.ahrq.gov/sites/default/files/assessing-the-risk-of-bias_draft-report.pdf, accessed on 10 October 2024) for assessing the risk of bias in cross-sectional studies. Quality scores were assigned based on the following checklists: (1) Source of information clearly defined, (2) Specified criteria for selecting sampling location, (3) Procedural controls included, (4) specified sampling period, (5) Assay methods clearly described, (6) Indicated method for quality assurance, (7) Outcome indicators (e.g., number of MPXV positive samples or genomic DNA concentration) clearly indicated, (8) PCR assessment for potential inhibition specified, (9) Describe the recovery efficiency of the concentration protocol, (10) described how missing data were managed, (11) Identified the potential biases in the study. The quality of an article was rated as “low quality” if it scores 0–4 points (C), “moderate quality” if it scores 5–8 points (B), and “high quality” if 9–11 points (A).

### 2.6. Data Synthesis

The information collected from the database comprises published articles that were combined and the meta-analysis was performed using RStudio version 4.3.1. The overall prevalence of the positivity rate of mpox viral PCR in different specimens was estimated. A random-effects model developed by Dersimonian and Laird [15] was used for the quantitative analysis. The 95% confidence intervals for the proportions reported in each study were calculated. The Cochran’s Q test and the *I*^2^ statistic were used to assess the between-study heterogeneity. Heterogeneity was measured using the *I*^2^ statistic, where a value of >50% or a *p*-value of <0.05 indicates significant heterogeneity and a *p*-value < 0.05 was a sign of statistical heterogeneity in the Cochran’s Q test [16]. To assess the impact of the covariates, a subgroup analysis was performed according to the region, epidemiological year, PCR methods, sampling strategy, and sample concentration methods. This analysis employed Egger’s regression to determine the potential for publication bias. The presence of potential publication bias was considered when the *p*-value was less than 0.05.

## 3. Results

### 3.1. Baseline Characteristics

**Figure 1:**
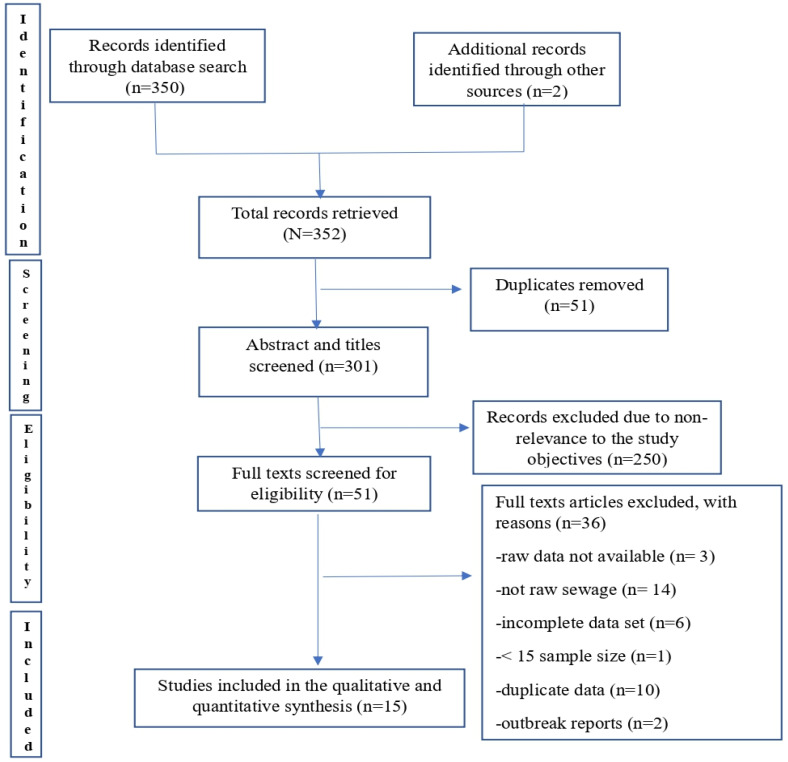
Flow diagram of included studies and retrieval processes.

A total of 352 publications were identified via electronic database and citations search. Fifty-one duplicates were removed after the initial screening. Further, the screening of article titles and abstracts resulted in the exclusion of 250 irrelevant records. A total of 51 potentially relevant articles were assessed for eligibility, out of which 36 were further excluded due to reasons that included unavailability of raw data, wastewater type not raw wastewater, incompleteness of data set, data duplications, case-based epidemiological reports, and sample size inadequacy. The remaining 15 peer-reviewed articles with satisfactory qualities were included in the qualitative and quantitative synthesis (Figure 1 and Table 1) [9,17,18,19,20,21,22,23,24,25,26,27,28,29,30]. The studies recorded moderate (10/15) to high-quality (5/15) assessment scores. Table 1 depicts the characteristics of included individual studies and quality assessment scores. According to the United Nations demarcation, there were 6 studies from North American countries, 7 from Europe and Central Asia, and 1 each from countries in Latin America and the Caribbean and the East Asia and Pacific region. Most studies (n = 13) were conducted in 2022 while only 2 studies were conducted in 2023. Thirteen of the studies obtained composite samples, while 1 obtained a grab sample and both grab and composite samples. The methods employed for viral recovery from wastewater included adsorption extraction (n = 2), centrifugation (n = 6), ultracentrifugation (n = 1), vacuum filtration (n = 1), PEG precipitation (n = 1), PEG precipitation and adsorption elution method combined (n = 1), and unknown (n = 3). Most studies employed real-time quantitative PCR (qPCR) (n = 11) while only 4 used the digital droplet PCR (ddPCR) for genomic DNA detection and quantification. Ten studies obtained both wastewater and clinical samples data, but only a part of them provided a complete data set for computing correlation coefficients while the others described phylogenetic results for MPXV-positive samples.

### 3.2. The Overall Pooled Positivity Rate of the Mpox Virus in Wastewater

**Figure 2:**
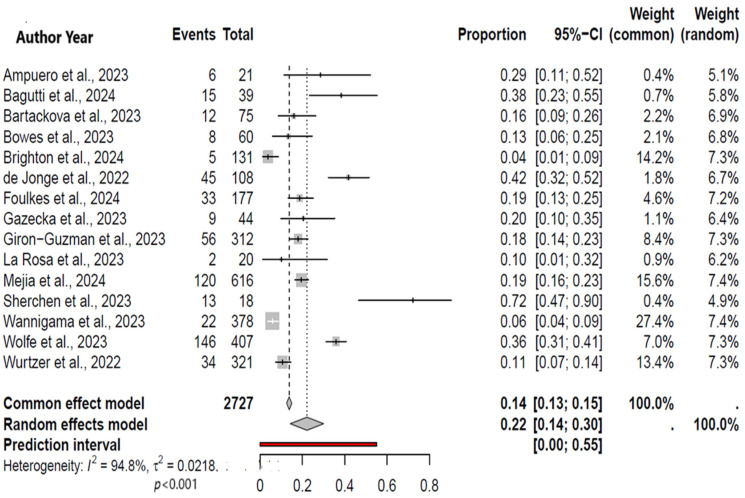
A forest plot showing the pooled rate of MPXV positivity in wastewater [9,17,18,19,20,21,22,23,24,25,26,27,28,29,30].

Out of 352 studies, 15 were eligible for a meta-analysis. Among included studies, the individual positivity rate of the MPXV ranged from 1% to as high as 27%. The meta-analysis included a total number of 2727 wastewater samples (526 showed positivity), revealing an overall MPXV positivity rate of 22% (95% CI: 14−30%; *I*^2^ = 94.8%) (Figure 2). Of note, the analyzed samples are not reflective of the overall specimens included in these studies (Table 1) since only a minority met the inclusion criteria.

#### 3.2.1. Subgroup Analysis Based on Region of Studies

**Figure 3:**
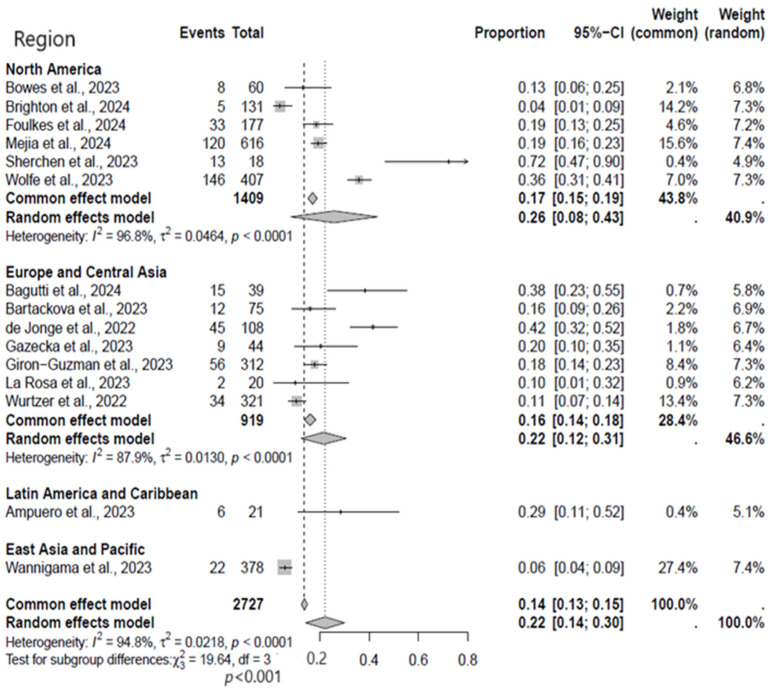
Subgroup analysis forest plot of MPXV positivity in wastewater based on region of studies [9,17,18,19,20,21,22,23,24,25,26,27,28,29,30].

A subgroup analysis was performed to identify regions with significant evidence of MPXV circulation in the environment. The stratified analysis showed that the rate of MPXV detection was substantially higher in studies from North America (26%, 95% CI: 8–43%) than in studies from Europe and Asia (22%, 95% CI: 12–31%) (Figure 3). At the same time, both regions exhibited a statistical heterogeneity.

#### 3.2.2. Subgroup Analysis Based on Epidemiological Year

**Figure 4:**
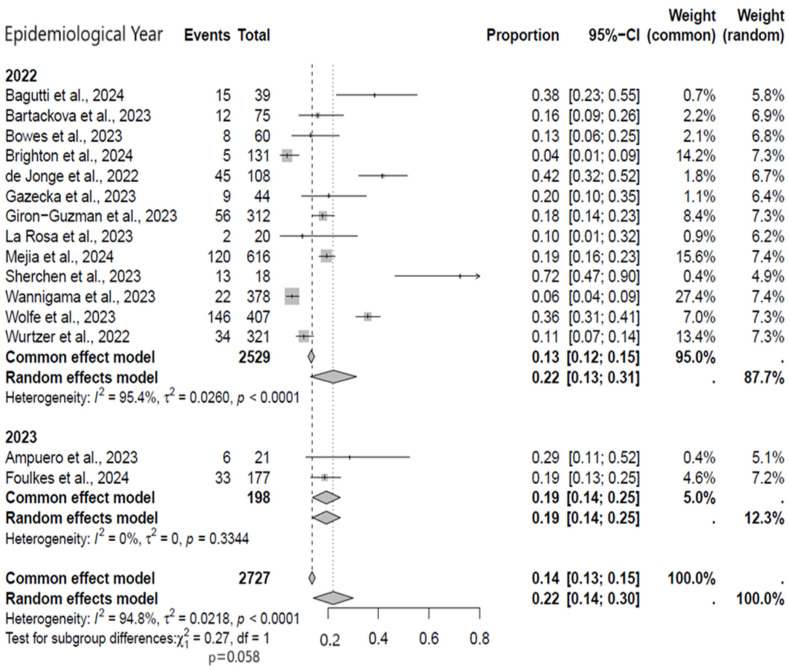
Subgroup analysis forest plot of MPXV positivity in wastewater based on epidemiological year [9,17,18,19,20,21,22,23,24,25,26,27,28,29,30].

In the subgroup analysis for the epidemiological year, most of the studies included were published in the year 2022. The meta-analysis for the year 2022 included a total number of 2529 wastewater samples (497 samples showed positivity) while the year 2023 comprised 198 samples (39 showed positivity) (Figure 4). When comparing the frequency of viral detection, this was significantly higher in the year 2022 studies (22%, 95% CI: 13–31%) compared to 2023 (19%, 95% CI: 14–25%). Overall, there was no statistically significant heterogeneity in the year 2023. This is expected because the positivity rate is far below what was recorded in the preceding year.

#### 3.2.3. Subgroup Analysis Based on Sample Concentration Methods

**Figure 5:**
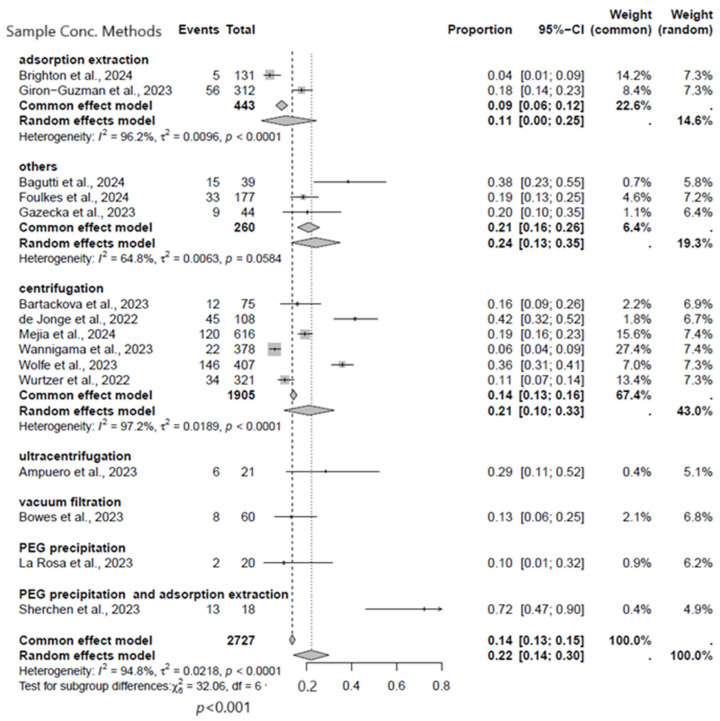
Subgroup analysis forest plot of MPXV positivity in wastewater based on sample concentration methods [9,17,18,19,20,21,22,23,24,25,26,27,28,29,30].

In this subgroup analysis, six different sample concentrations and three unknowns (grouped as others) were utilized in the studies that met the inclusion criteria. When comparing the frequency of sample concentration methods, significantly higher rates of MPXV were detected with unknown concentration type (24%, 95% CI: 13–35%) and in centrifugation (21%, 95% CI: 10–33%) than other types of sample concentration methods (Figure 5).

#### 3.2.4. Subgroup Analysis Based on Sampling Strategy

**Figure 6:**
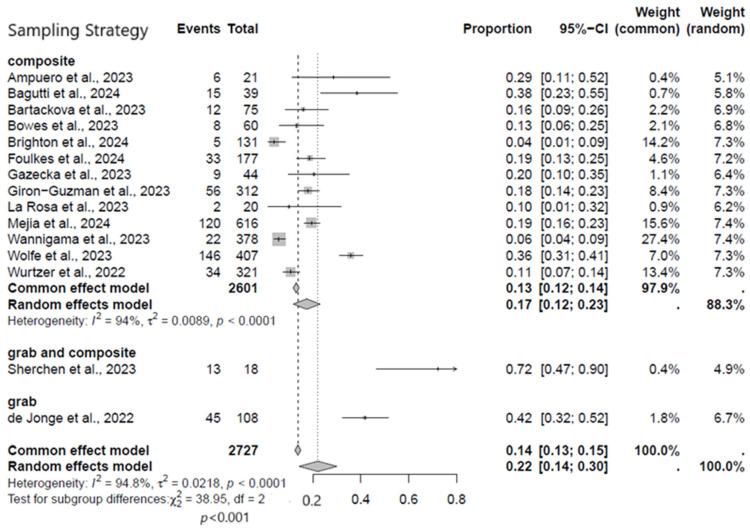
Subgroup analysis forest plot of MPXV positivity in wastewater based on sampling strategy [9,17,18,19,20,21,22,23,24,25,26,27,28,29,30].

Additionally, considering the relevance of the sampling strategy, we performed a subgroup analysis to assess the best type of sampling adopted in the study that met the inclusion criteria. For instance, sampling is a method that allows researchers to infer information about a population based on results from a subset of the population, without having to investigate every individual. Since most of the studies did not meet the inclusion criteria, we decided to perform a subgroup analysis based on the sampling strategy adopted in the studies that we used for the meta-analysis. Overall, the studies included in the meta-analysis adopted three different types of sampling strategies, of which a total number of 2727 were composite samples with an overall MPXV positivity rate of 17% (95% CI: 12−23%; *I*^2^ = 94%) (Figure 6).

#### 3.2.5. Subgroup Analysis Based on Sample PCR Methods

**Figure 7:**
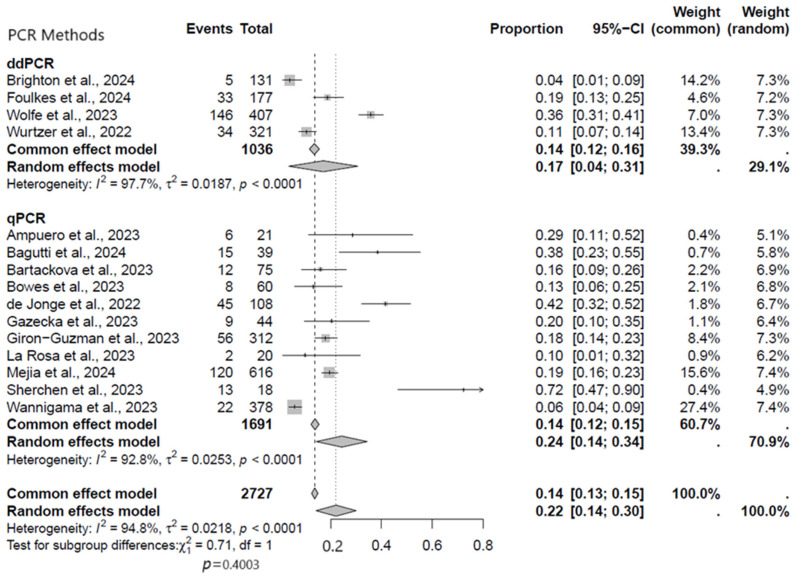
Subgroup analysis forest plot of MPXV positivity in wastewater based on PCR methods [9,17,18,19,20,21,22,23,24,25,26,27,28,29,30].

On the basis of PCR methods, we performed a subgroup analysis to see which methods of PCR performed best in the current meta-analysis. In terms of viral DNA detection rates, studies that employed qPCR appeared to have performed better compared to the ddPCR platforms. In the subgroup analysis, the pooled rate of MPXV detection across different regions was significantly higher using the qPCR studies (24%, 95% CI: 14–34%) than in ddPCR studies (17%, 95% CI: 4–31%) (Figure 7).

#### 3.2.6. Publication Bias

**Figure 8:**
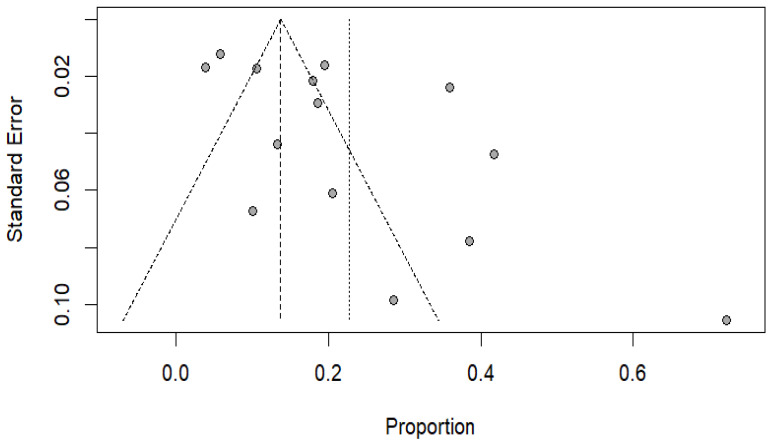
Funnel plot with pseudo 95% CIs for detection of publication bias among included studies.

The outcomes of Egger’s regression test and the irregularity in the funnel plot demonstrated notable publication bias within the studies encompassing knowledge that was incorporated in this meta-analysis (bias: 4.5685 (SE = 1.9154); t = 2.39, df = 12, *p*-value = 0.0344), thus rejecting the null hypothesis of symmetry. Thus, it can be shown that the asymmetry in the results and in the image explains the wide differences in the reported positivity values (Figure 8).

### 3.3. Comparison Between MPXV Detection Rates in Wastewater and Incidence Cases of Mpox

The establishment of correlation was based on the analysis of individual studies where both data were available rather than pooling the different studies. This became necessary because of the variation in the sampling periods in different studies. Pearson’s correlation analysis was carried out to assess for any relationship between MPXV detection in wastewater and the number of new Mpox cases. Of the five studies [18,19,25,26,30] with information on both viral detection rates in wastewater and cases of mpox during the study period, only in Gazecka et al. [25] was MPXV detection in wastewater not correlated with incidence cases (Supplementary Figure S1a–e).

## 4. Discussion

Mpox cases continue to be reported globally, though the actual burden of the disease in the general population is likely underestimated since the proportion of asymptomatic or unreported cases is precluded in the current clinical-based surveillance studies. Thus, wastewater-based epidemiology (WBE) has been suggested as a potential epidemiological monitoring tool for timely prevention and response to MPXV disease complementary to case-based surveillance strategies. According to a recent WHO report, a total of 115,101 confirmed cases of mpox and 255 deaths were reported in all six WHO regions between January 2022 and October 2024. In October 2024 alone, 3233 new confirmed mpox cases were reported worldwide, majority of whom were in Africa (71.0%), followed by Western Pacific region (11.6%) and Americas (9.8%). As of December 2024, most countries in Africa including Democratic Republic of the Congo, Burundi and Uganda have ongoing outbreaks [4] and three others (Zambia, Mauritius, and Zimbabwe) have recorded their first case [1]. On 25 November 2024, WHO recognized the potential benefit of WBE of MPXV and provided the practical interim guidance on its application as part of multimodal surveillance for mpox worldwide [31]. Thus, we summarized the surveillance data from different wastewater-based studies on MPXV, to provide the pooled prevalence of viral positivity rates, correlate wastewater surveillance data with incidence data, and ultimately provide empirical evidence that could guide future research.

In the current study, an overall MPXV detection rate of 22% (95% CI: 14–30%) in wastewater was recorded, an observation suggesting a high prevalence of the disease in the human population. This finding supports the body of knowledge that MPXV are shed in feces or urine from symptomatic and/or subclinical cases [12,32]. In a recent epidemiological study by Yinda et al. [33], the ability of MPXV to survive for weeks in wastewater was demonstrated, corroborating the overall level of detected viral concentration despite the extensive dilution in wastewater and toxicity. Although MPXV is primarily transmitted through close physical contacts, such as the exchange of body fluids, skin-to-skin contact, and contact with respiratory droplets, the frequent shedding in feces and the confirmation in the study of high detection rates in wastewater from pooled studies drawn from different parts of the world, support the potential use of WBS as a tracking tool for MPXV in wastewater environments. Considering the existence of other shedding pathways, the observed prevalence of MPXV in wastewater could only be a part (despite being significant) of the actual global prevalence. Although the potential public health risk for environmental transmission of MPXV is yet to be fully established, a recent study by Ampuero et al. [21] confirms the infectivity of the MPXV virus that was recovered from Chilean wastewater. Additionally, the ability of MPXV to retain its infectiousness for weeks in untreated wastewater [33] suggests the potential infection risk associated with wastewater exposure.

Meta-analytic findings from pooled studies from countries in North America indicated a higher MPXV detection rate in wastewater relative to countries from Europe and Central Asia. The differential rates could be explained, in part, by the different viral concentration strategies, nucleic acid extraction methods, molecular detection modalities, influence of environmental factors on virus stability, or disparities in the prevalence of mpox in the general population. From an epidemiological perspective, it was surprising that no documented reports of wastewater-based MPXV detection were available from African countries such as the Democratic Republic of Congo, Burundi, Central African Republic, Rwanda, and Nigeria despite bearing the significant burden of mpox [3]. According to Wannigamma et al. [26], the lack of an organized and centralized wastewater infrastructure in these poor socioeconomic settings may partly explain this observation. Of note, a knowledge gap exists between the MPXV virus circulating in the human population and environmental reservoirs in African regions, thus WBS to shed light on these discrepancies is warranted by future studies in the region. Interestingly, the tracing of mpox viral DNA in non-sewered wastewater by Wannigamma et al. [26] strongly suggests the feasibility and applicability of wastewater-based surveillance for MPXV in poor resource countries/regions with no established sewer systems. In the developing African countries with the brunt of the mpox disease burden, the limited access to healthcare systems and lack of routine surveillance may underestimate the actual disease burden. Notably, the lack of wastewater surveillance which provides real-time, actionable data from different individuals irrespective of symptoms represents a crucial gap in the region’s disease control efforts as there may be the possibility of silent virus transmission when patients fail to seek medical attention or report suspected cases due to the social stigma associated with mpox. Thus, by increasing the resources for wastewater surveillance in African regions and integrating it with clinical testing, the concerning trends in the spread of mpox in the general population as recently posited by the National Center for Infectious Diseases (NICD) [34] and WHO [1] could be checked.

The significantly high occurrence of MPXV in wastewater from North America could be a reflection of the high vaccine hesitancy that has been reported in the region [34], especially in the United States where most of the wastewater-based studies are available. To curtail the disease outbreak in 2022, a live attenuated vaccinia virus vaccine developed by Jynneos, Imvamune, and Imvanex was made available to populations [35]; however, only 23% of the at-risk population in the Americas region had received full doses of the vaccine as of January 2024, with incident cases being the population with partial or no history of the Jynneos vaccine [36]. In the Asian countries with lower MPXV detection in wastewater, refusal to be vaccinated by health professionals has been reported to occur less frequently regardless of the relatively low literacy rates [37]. The findings from this study suggest that, in addition to monitoring the mpox trends via wastewater surveillance, it may be possible to predict the effectiveness of the mpox vaccine in a fully vaccinated population. Our study revealed a significantly low MPXV detection rate in wastewater in 2023 compared to the preceding year, despite the increasing trends in the global prevalence of mpox [1]. The probable reason could be the smaller number of included studies compared to the 2022 epidemiological year. Thus, for a better understanding of the epidemiological benefit of wastewater-based surveillance for MPXV, continuous and worldwide monitoring of the pandemic strains would be beneficial.

This meta-analysis showed that >90% of studies implemented the composite sampling strategy, boosting our confidence in the reliability of the samples included in the review. However, the very few studies that employed the grab sampling approach make it difficult for a comparative analysis of the relative benefit of one sampling approach over the other. Notwithstanding, preference for the composite sampling approach has been demonstrated as it can catch variation of the wastewater over time, thereby providing a better representative snapshot of the community of interest as compared to the grab sampling strategy, which is more biased regarding the material collected at a particular point in time [38,39]. Despite the large uniformity in the sampling strategies, we did observe differences in sample concentration methods and nucleic acid extraction across various studies that could impart the viral recovery rates and DNA concentration ranges. A reasonably high prevalence of MPXV in wastewater was recovered via centrifugation concentration procedures as compared to the surface adsorption extraction modalities. Although there are currently no approved general concentration procedures for MPXV monitoring in wastewater, comparative experiments involving centrifugation of wastewater to separate the aqueous phase from the biosolids, followed by DNA extraction from the latter have been shown to produce better results than the former [29] and, by extension, other methods targeting the aqueous portion [23]. For a better understanding of the observed disparities ranging from viral concentration to recoveries, there is a need for regular sampling and optimization of the wastewater-based surveillance methods to ascertain the most robust and reliable procedure for capturing low-abundance viruses in the wastewater matrix. The findings of generally high Ct values for MPXV DNA amplification in the included studies, though maybe a reflection of a low MPXV case count, could also suggest the need for an overall improvement in MPXV recovery and detection platforms.

This meta-analytic study shows that the real-time qPCR mostly used for MPXV DNA detection in different studies significantly detected higher viral concentration compared with that of the ddPCR platform (25% vs. 17%). The observed qPCR MPXV positivity rates in wastewater environments in this study, even though significantly high, may not be fully representative of the actual global shedding rates because of its technical limitations. According to Ding et al. [40], the quantitative accuracy of the widely used qPCR platform for genomic surveillance in wastewater is usually limited by low virus concentration, the presence of organic inhibitors, and the co-occurrence of viral genotypes. Thus, there exists the possibility of false positive or false negative results which has been documented [23]. Findings from this study showed that the application of ddPCR for wastewater surveillance remains limited as only 29% of studies adopted the method. Although, ddPCR methodology has surpassed the inherent limitations of qPCR and has proven to be a preferred alternative platform for SARS-CoV-2 detection and by extension, other viruses in wastewater [40], wider studies on different wastewater matrixes from different laboratories are needed to evaluate the absolute quantitative performance of the ddPCR for MPXV and its comparability with the qPCR.

Interestingly, the finding of a positive correlation between several wastewater-based studies of MPXV and case detection pinpointed the complementary role of WBS to case-based surveillance for guiding the public health response towards the mpox outbreak. In other studies [9,28,41], where the actual incidence cases could not be extracted for our review, a significant correlation was demonstrated between wastewater and clinical case data, supporting our hypothesis that detectability of MPXV DNA in wastewater is possible when MPXV-infected individuals are present within the contributing sewer shed. In one study [27] where correlation could not be established despite the high MPXV detection in wastewater, the social stigma associated with mpox and the resultant effect of being missed out on clinical case testing was suggested as possible reasons for the mpox cases being underreported. Gazecka et al. [25] also posited that the lack of correlation between MPXV detection in wastewater and the number of new rates of hospitalizations in Poland was a result of the MPXV-infected individuals not being identified by the public health authority. The lack of data in terms of shedding rate per individual at various stages of the disease development, warrant its investigation in a future study. In addition to human factors, viral aspects such as the decay and dynamics of MPXV shedding that impact MPXV concentration in wastewater [26] could be the plausible reason for the negative relationships between both trends.

### 4.1. Limitations

The current study has certain limitations. Firstly, the lack of wastewater-based studies on MPXV detection from countries in Africa currently with the highest burden of mpox may have impacted the overall MPXV prevalence in wastewater estimated in the study. Considering the absence of WBE data from the endemic regions with no surveillance reports, the current global prevalence rate may be higher than estimated as data from the region, if available, could have contributed more to the overall viral load concentration. Secondly, the detection of a significantly high publication bias suggests that the results of this study are probably impacted by the selective publication of studies with positive or significant results. The implication is that the true effect size is likely overestimated since studies with negative or statistically non-significant findings may not be published or enlisted in the review. Thirdly, the zoonotic nature of MPXV makes it difficult to entirely relate the overall genomic data to human sources as MPXV released from animal reservoirs to sewage systems may complicate wastewater surveillance data interpretation for human cases. Nevertheless, a one-health approach study by Tiwari et al. [12] indicates that the presence of MPXV in wastewater whether from animals or the environment can pose an infection risk to humans. Fourthly, the limited number of studies reporting both MPXV detection rates in wastewater and the number of newly diagnosed mpox cases during the study period makes it difficult to clearly understand the true correlation between viral occurrence in wastewater and trends of clinical cases. Nevertheless, findings from this study are important as it has not only identified areas of research gaps but also provided relevant epidemiological data that could support the utility of wastewater surveillance for MPXV detection.

### 4.2. Conclusions

The overall findings from this study suggest that wastewater surveillance for MPXV has emerged as a viable surveillance strategy to monitor the viral occurrence and spread even when case numbers are low or no clinical cases are known to be present. The fewer numbers of reported studies post the year of MPXV re-emergence calls for concern, as ongoing monitoring of MPXV in wastewater is necessary to understand the actual population excretion rates and prioritization of resource allocation by public health authorities. The findings of a strong positive correlation between MPXV detection in wastewater and the number of symptomatic patients within catchment areas in most studies support the value of wastewater-based surveillance strategies. Clinical case testing within public health is currently limited by the social stigma associated with mpox, thus the ability of wastewater surveillance to capture viral shedding from unreported cases as anonymous samples supports that WBE can potentially predict with high accuracy the development and progress of the current MPXV disease outbreak. Nonetheless, the high heterogeneity observed among included studies suggests the need for further improvement and uniformity in viral recovery and detection platforms.

## Figures and Tables

**Table 1:** General characteristics of included studies.

Author	Country	Region	Period of Sampling	Sampling Strategy	Sample Size	Sample Concentration	PCR Methods	Recovery (%)	Ct Range	DNA Conc Range (gc/L)	MPXV Positive Samples	Proportion	Quality Score (A = 9–11) (B = 5–8) (C = 0–4)
Brighton et al. [17]	United States	North America	March–June 2022	Composite	131	Adsorption extraction	ddPCR	NR	NR	0–1.7 × 10^3^	5	3.8	B
Bagutti et al. [18]	Switzerland	Europe and Central Asia	July–August 2022	Composite	39	Others	qPCR	NR	34–36	5 × 10^2^–1.5 × 10^3^	15	38.5	A
Foulkes et al. [19]	United States	North America	March–June 2023	Composite	177	Others	ddPCR	NR	NR	NR	33	18.6	B
Mejia et al. [20]	Canada	North America	June–September 2022	Composite	616	Centrifugation	qPCR	NR	31–40	3 × 10^3^–2 × 10^4^	120	19.5	B
Ampuero et al. [21]	Chile	Latin America and Caribbean	April–September 2023	Composite	21	Ultracentrifugation	qPCR	NR	NR	3.5 × 10–2.2 × 10^3^	6	28.6	A
Giron-Guzman et al. [22]	Spain	Europe and Central Asia	May–August 2022	Composite	312	Adsorption extraction	qPCR	NR	34.3–44.3	2.2 × 10^3^–8.7 × 10^4^	56	17.9	B
Bowes et al. [23]	United States	North America	July–October 2022	Composite	60	Vacuum filtration	qPCR	22 ± 10	38.1–46.9	NR	8	13.3	A
La Rosa et al. [24]	Italy	Europe and Central Asia	May–August 2022	Composite	20	PEG precipitation	qPCR	NR	38.37–40.18	NR	2	10	B
Gazecka et al. [25]	Poland	Europe and Central Asia	July–December 2022	Composite	44	Others	qPCR	NR	38.25 ± 1.15	NR	9	20.5	B
Wannigama et al. [26]	Thailand	East Asia and Pacific	May–August 2022	Composite	378	Centrifugation	qPCR	NR	NR	1.1 × 10^4^–9.3 × 10^4^	22	5.8	A
Wolfe et al. [9]	United States	North America	June–August 2022	Composite	407	Centrifugation	ddPCR	>10	NR	9 × 10^2^–2 × 10^4^ gc/g	146	35.9	B
Sherchen et al. [27]	United States	North America	July–September 2022	grab and composite	18	PEG precipitation and adsorption extraction	qPCR	NR	NR	NR	13	72.2	B
Wurtzer et al. [28]	France	Europe and Central Asia	April–July 2022	Composite	321	Centrifugation	ddPCR	75	NR	2 × 10^3^–4 × 10^4^	34	10.6	B
de Jonge et al. [29]	Netherlands	Europe and Central Asia	May–July 2022	Grab	108	Centrifugation	qPCR	NR	35.7–42.9	NR	45	41.7	A
Bartackova et al. [30]	Czech Republic	Europe and Central Asia	August–November 2022	Composite	75	Centrifugation	qPCR	NR	NR	NR	12	16.0	B

Note: qPCR = real-time quantitative PCR, ddPCR = digital droplet PCR, PEG = polyethylene glycol, NR = not reported, CT = cycle threshold, MPXV = Monkeypox virus.

## Data Availability

Data used in the study are included in the article and its Supplemental Files.

## Supplementary Materials

The following supporting information can be downloaded at: https://www.mdpi.com/article/10.3390/v17030308/s1, Figure S1a–e. Relationships between MPXV detection in wastewater and incidence mpox cases.
