# Supplementary material for: Monkeypox Virus Occurrence in Wastewater Environment and Its Correlation with Incidence Cases of Mpox: A Systematic Review and Meta-Analytic Study
Source: Viruses. 2025 Feb 24;17(3):308. doi: 10.3390/v17030308 (PMC11945618; doi:10.3390/v17030308)
Supplement: Supplementary file 1 [file viruses-17-00308-s001.zip › viruses-3480639-supplementary.pdf]

Figure S1(a-e). Relationships between MPXV detection in wastewater and incidence mpox cases.

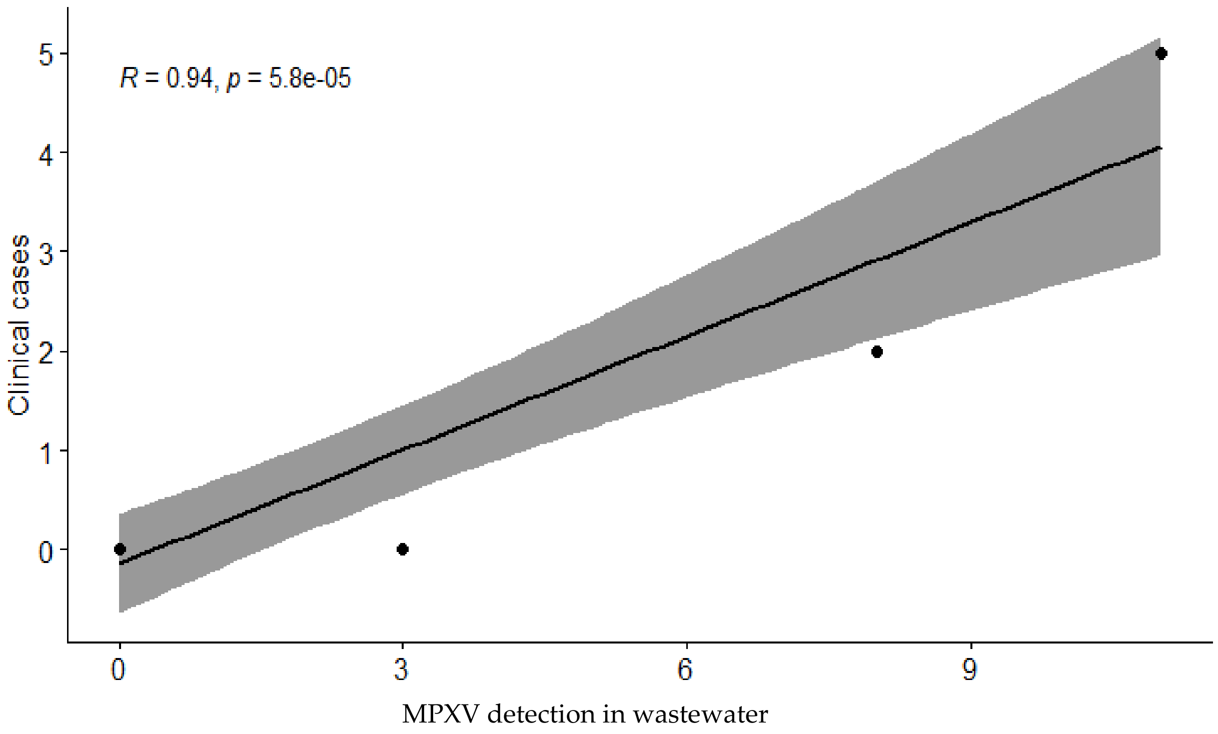

(**a**) Wannigama et al., 2023

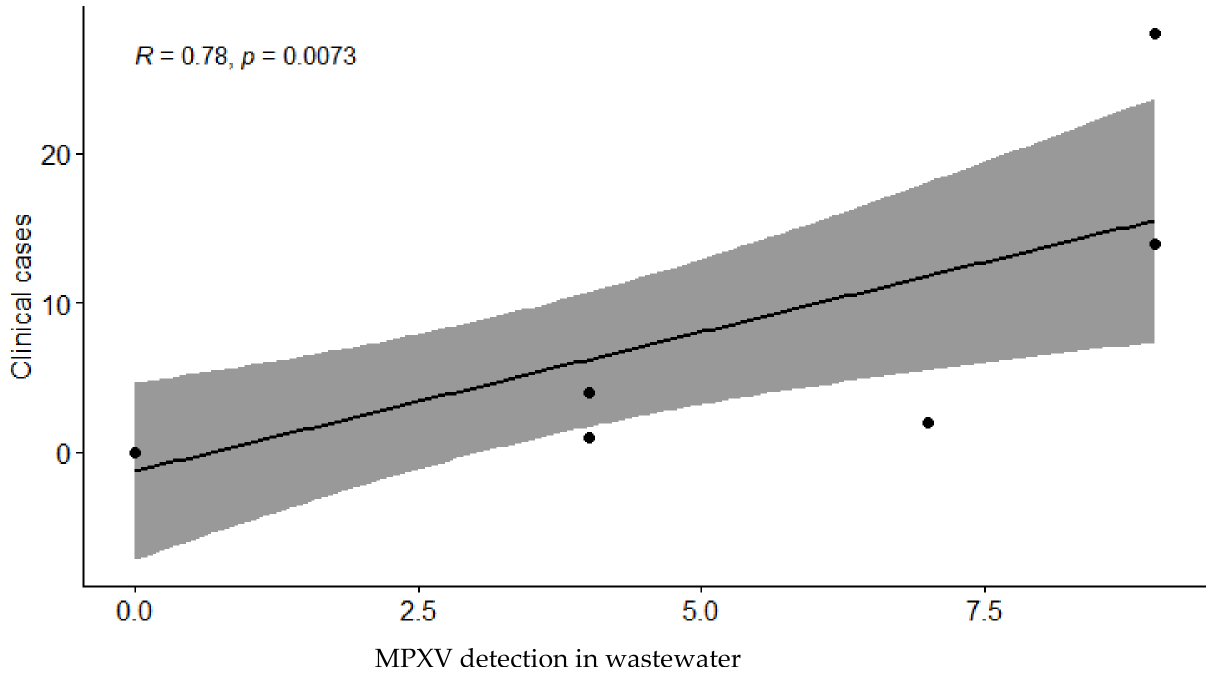

(**b**) Foulkes et al et al., 2024

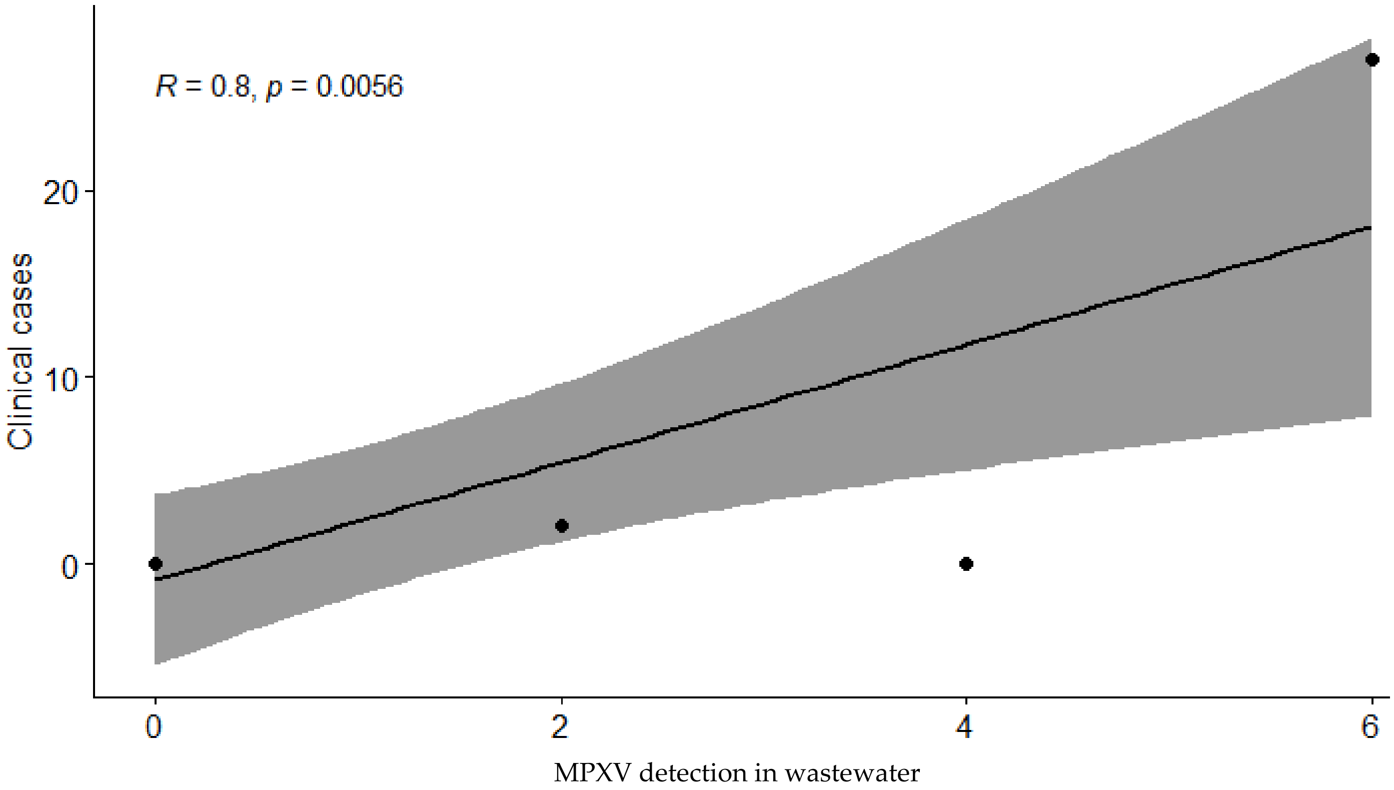

(**c**) Bactackova et al., 2023

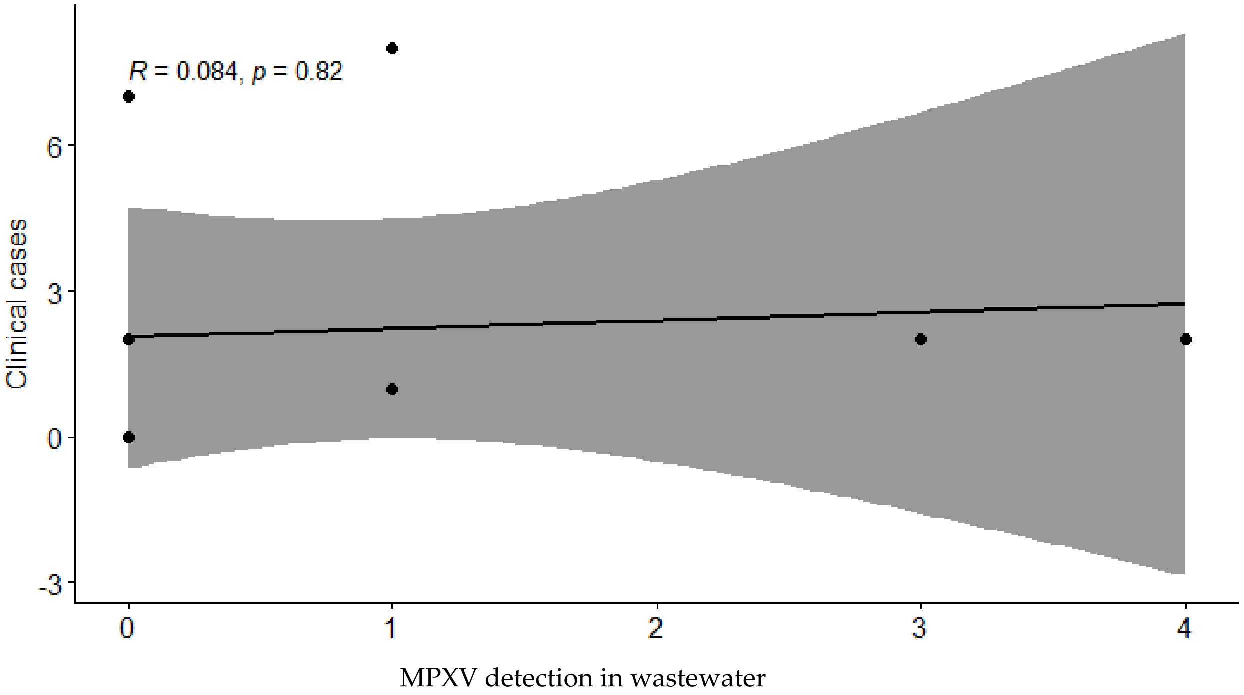

(**d**) Gazecka et al., 2023

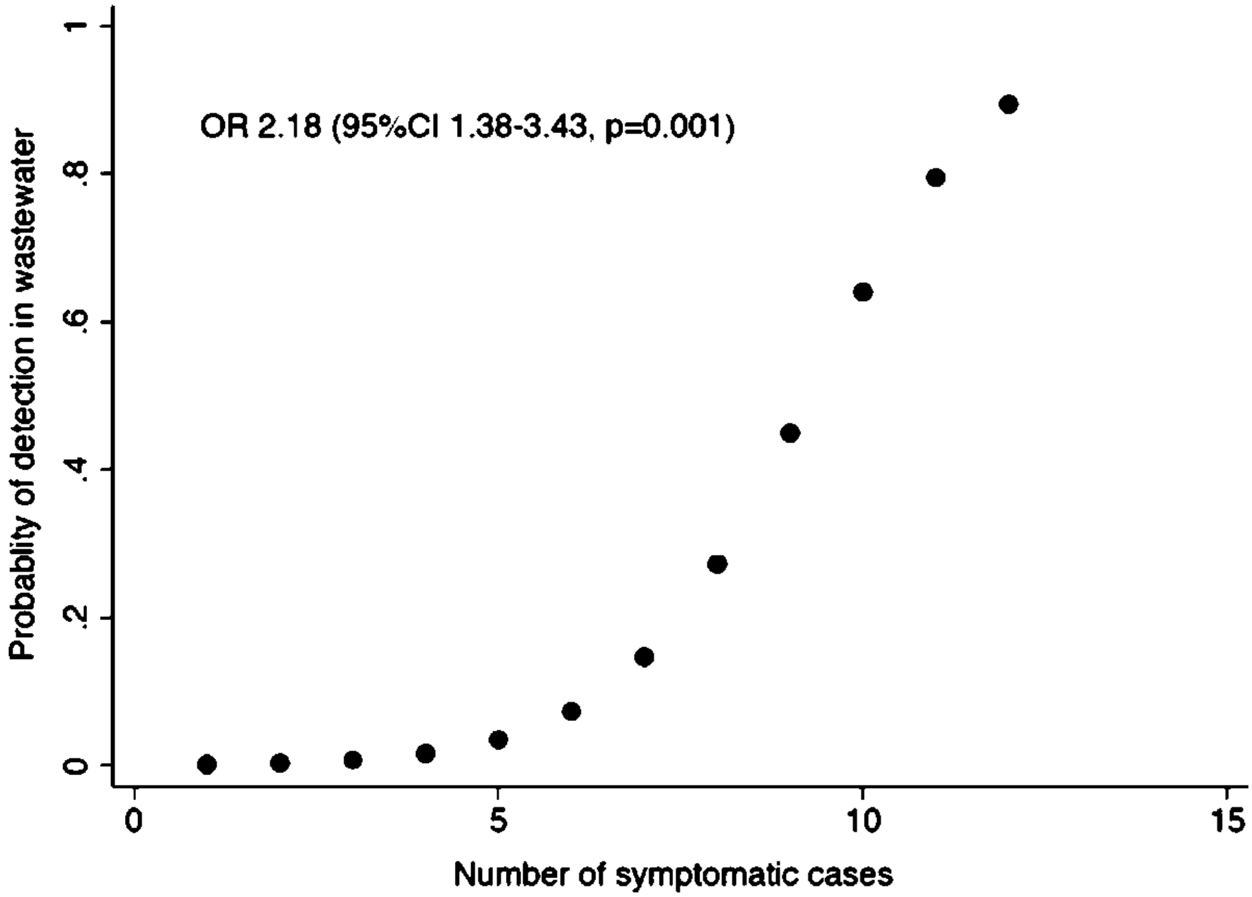

(**e**) Bagutti et al., 2024
